# Carbon Fiber Reinforced Composites: Study of Modification Effect on Weathering-Induced Ageing via Nanoindentation and Deep Learning

**DOI:** 10.3390/nano11102631

**Published:** 2021-10-06

**Authors:** Georgios Konstantopoulos, Dionisis Semitekolos, Elias P. Koumoulos, Costas Charitidis

**Affiliations:** 1 RNANO Lab—Research Unit of Advanced, Composite, Nano Materials & Nanotechnology, School of Chemical Engineering, National Technical University of Athens, Zographos, GR-15773 Athens, Greece; gkonstanto@chemeng.ntua.gr (G.K.); diosemi@chemeng.ntua.gr (D.S.); charitidis@chemeng.ntua.gr (C.C.); 2 Innovation in Research & Engineering Solutions (IRES), Boulevard Edmond Machtens 79/22, 1080 Brussels, Belgium

**Keywords:** carbon fibers, composites, nanoindentation, impact behavior, interphase, artificial intelligence, neural networks, deep learning

## Abstract

The exposure of carbon-fiber-reinforced polymers (CFRPs) to open-field conditions was investigated. Establishment of structure–property relations with nanoindentation enabled the observation of modification effects on carbon-fiber interfaces, and impact resistance. Mapping of nanomechanical properties was performed using expectation-maximization optimization of Gaussian fitting for each CFRPs microstructure (matrix, interface, carbon fiber), while Weibull analysis connected the weathering effect to the statistically representative behavior of the produced composites. Plasma modification demonstrated reduced defect density and improved nanomechanical properties after weathering. Artificial intelligence for anomaly detection provided insights on condition monitoring of CFRPs. Deep-learning neural networks with three hidden layers were used to model the resistance to plastic deformation based on nanoindentation parameters. This study provides new assessment insights in composite engineering and quality assurance, especially during exposure under service conditions.

## 1. Introduction

Carbon-Fiber (CFs)-reinforced Polymers (CFRPs) are on the peak of their development, and are expected to be utilized massively in aerospace, automotive, and construction markets as substitutes to metal compartments [1,2,3,4,5,6,7,8,9,10]. Their advantages over other end-use materials are their low specific density and mechanical robustness, which have been highly valued by the research community [2,3,5,7]. On the spotlight of their recyclability concerns, the incorporation of thermoplastic matrices attracts interest, in order to reduce the end-to-end ecological footprint [10,11,12,13]. An alternative way to enable an eco-friendlier application of CFRPs, and also to increase their added value, is by extending their service life, which can be (nano-)engineered by modification of CFs’ surface chemistry [9,14]. Chemical modification at nanoscale level can prevent brittle failure of composites, and improve mechanical properties during service life, as composite performance is dominated by the interface [8,15,16,17,18,19]. Mechanical interlocking can be efficiently improved by applying green high-throughput technologies, such as plasma and electropolymerization [10]; enhanced fiber-matrix wetting functions twofold; by facilitating load transfer in the interface, and; by preventing moisture-induced degradation [15,19,20,21]. To date, only anodic oxidation, plasma oxidation, and polymer grafting have been economically viable for industry [21,22]; the widening effect of interphase thickness commonly accounts for 100–500 nm upon surface modification treatment of CFs [23].

Exposure of CFRPs to an energy-intensive environment, including a thermal gradient and UV irradiation, which both initiate oxygen-induced free-radical reactions, results in chemical and physical ageing [1,2,20]. Chemical ageing could have a beneficial impact to CFRPs by post-curing—especially for treatment below the onset temperature of glass transition [3,24]—or negatively affect the composite by oxidatively degrading the interface and matrix, which leads to chain scission and evolution of volatiles [1,2,5,24]; the latter emphasizes the impact of engineering to reduce the available spots for degradation, by increasing the strength of interphase binding and preventing delamination upon loading or thermal relaxation, which introduces spaces that allow humidity to penetrate. On the other hand, physical ageing effects can be envisioned by microcrack generation, due to the different expansion coefficients of the fiber and matrix [4,15]. The increase in the exposed interface in the degradative oxygen atmosphere is reported to lead to shortening of the composite lifetime [1,24,25]. UV exposure of CFRPs in humid environments has been linked with both interlaminar shear strength and elastic-modulus reduction in the composite [2], while UV wavelength energy (300–415 kJ/mol) is associated to epoxy-matrix depolymerization [15,20] and interface shrinkage [16]. Modulus deterioration is mostly attributed to hydrolysis of the existing bonds in the interface and is related to matrix plasticization [8,15,20,26], which is the main cause of brittle failure [16,20]. These phenomena are mostly determined by the exposure duration [2,20] and number of cycles, to emulate cyclically applied environmental factors during their life-cycle [2]. Progress of ageing is observed at a first glance in the outer layer, and thus a “skin” layer is formed that prevents shrinkage and leads to residual stress creation in the interface [5]. Nanoindentation appears as an essential tool to determine the interface performance after ageing [8] at local level, since the interface is significantly affected by thermal expansion of the constituent phases [15].

Condition monitoring with unsupervised machine learning enables a multi-level architectural view, which is a key feature in fingerprint/topology detection of a device or component [27], such as motors [28] and wind turbines [29], and can be correlated to anomaly detection [28,30]. Attributes of the generated clusters are described by the calculation of key inter- and intra-cluster distances and the mean square error that organize data to clusters [31]. Thus, cluster attributes can be connected to a microstructure or a failure mode of an end-product [30,32]. In the case of CFRP engineering, the incorporation of modification treatments may alter the CFRP fingerprint by improving interfacial properties. Of special interest is to identify the alternations in a CFRP fingerprint upon exposure to ageing factors. The detection of a different fingerprint in aged CFRPs may be correlated to failure modes of corrosion and erosion and could be used for condition monitoring. Crack generation and propagation due to different thermal expansion coefficient of the matrix and CF phases, and brinelling during testing (i.e., indentation) can introduce irregularities in the identified mechanical properties [30], termed as anomalies. In this direction, it is highly desirable to identify the failure-mode characteristics in order to use data-acquisition techniques to detect these faults in a component fingerprint. This may be a key step to introducing unsupervised estimators and finally qualitatively assessing [28] ageing using nanoindentation testing and machine learning for prognosis.

In this work, CFRPs were manufactured using two different engineering approaches to reinforce the interphasial properties. The functionalized carbon fibers underwent atmospheric pressure plasma (APP) irradiation and electropolymerization with polymethacrylic acid (PMAA). Specimens were subjected to cyclic UV/condensation according to ASTM G154, which reproduces damage caused by sunlight, rain, and dew by exposing materials to automated cycles of UV radiation and water-vapor condensation. Samples were tested over a full period of 1000 h. Weathering was performed to monitor the ageing behavior upon modification in regards to mechanical properties and comparison with a pristine unmodified composite was performed. As enhanced wetting between the fiber and matrix may hinder ageing effects, a detailed characterization at nanoscale was performed, which was further supported by novel artificial intelligence tools. Nanoindentation was used in order to evaluate nanomechanical properties and interface performance using Gaussian fitting and an expectation-maximization algorithm. The nanomechanical properties were further analyzed and insights were obtained using Weibull analysis to ensure the statistical representation of the obtained results and correlate indentation modulus to defect density. Additionally, hardness-to-modulus ratio was used to reveal a stress-relief mechanism in composites, regarding the energy of deformation, in order to better understand the impact resistance of the manufactured composites. Finally, artificial intelligence was involved for anomaly detection. Deep learning was performed on instant-hardness data, using unsupervised-neural-network clustering for learning the normality in data, in order to identify and assess the anomalies based on mean square error values, which were introduced to the CFRP structure by weathering.

## 2. Materials and Methods

Carbon fiber fabric G0926 (5H Satin-HEXCEL Industries, Amesbury, MA, USA) is composed from TENAX HTA40 E13 6K on both warp and weft. The weight is evenly distributed in yarn directions 0° and 90° reaching a nominal weight of 375 g/m^2^. The resin system was purchased from HUNTSMAN Industries (Maple Shade, NJ, USA) and is composed of an epoxy resin (Araldite LY556), an anhydride hardener (Aradur 917), and an imidazole accelerator (DY 070) with a weight-mixing ratio of 100:90:0.5. All composites were manufactured via the vacuum-assisted resin transfer molding process.

### 2.1. Surface Functionalization of CFs

Air pressure plasma is a modification technique where the CF fabrics are placed on top of an insulative table under a carriage that produces plasma on the surface of CF, and supports the electrode. The plasma power was set at 500 W, with a carriage speed of 5.4 m/s and argon as carriage gas [33]; more details about plasma processing and composite CFRP performance have been published elsewhere [34,35]. The electropolymerization took place in an electrochemical cell with aqueous solution of 0.3 M MAA (Acros Organics, double distilled under reduced pressure, monomer), 0.4 M ZnCl_2_ (Acros Organics, Electrolyte), 10 mM N,N′-methyl-bis-acrylamide (Acros Organics, cross-linker). The electropolymerization lasted for 1 h and ran at a potential of −0.435 V (potential required to reduce the monomer).

### 2.2. Composites Manufacturing

**Figure 1:**
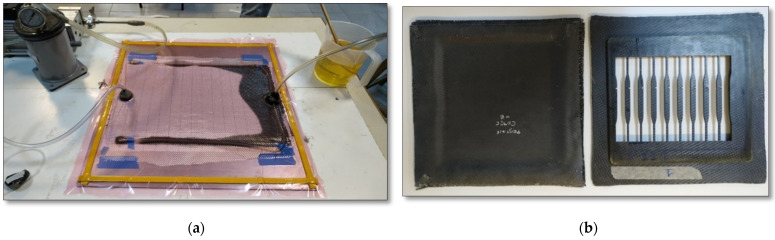
(**a**) VA-RTM for composite manufacturing, (**b**) Composite panel manufactured through VA-RTM (left) and scrap remained after cutting (right).

The manufacturing process that was used was vacuum-assisted resin transfer molding (VA-RTM). The set-up of the mould is presented in Figure 1.

The experimental procedure was as follows:

(a) Application of high-temperature release wax to the mold to achieve easy demolding.

(b) Eight layers of modified or unmodified fabric without resin were stacked dry in the mold.

(c) Placement of peel ply for successful removal of all the layers laid above the CF fabrics.

(d) Placement of infusion mesh, to facilitate the circulation of the resin during the infusion.

(e) Sealing with a vacuum bag, which prevents the air from entering the mold.

Consequently, the vacuum bag was pierced in two diametrically opposed sides. In the first one, the resin catch pot was connected, which in turn communicated with the vacuum pump. In this way, it was possible to draw all the air trapped between the layers of the fabric, while the trap was intended to protect the vacuum pump from the outgoing resin during the infusion process. In the second one, a beaker containing the resin was attached.

Once all the necessary connections were made, the air had to be pumped from the mold. The vacuum consistency was monitored by the manometer attached on top of the resin catch pot. Having secured the necessary vacuum, the clamp at the resin beaker was released, allowing the pump to direct the resin into the mold and through all the layers of fabrics, until it was finally seen to exit the trap. The clamps were closed, and the mold was transferred to the oven at 80 °C for 4 h for the curing process. Afterwards the mold was left to cool at room temperature, so the composite panels could be removed from the mold and then transferred to the furnace for another 4 h at 120 °C for the post-curing step. The panel-type composite materials were then cut into specimens via water jet CNC to the desired dimensions based on the standards for tensile testing. In Figure 1, the prepared composites are presented prior to and after cutting.

### 2.3. Weathering of CFRPs

The panels were tested in a Q-Lab weathering chamber (QUV) (Q-LAB EUROPE, LTD., Bolton, England) cabinet operating an accelerated weathering scheme as per ASTM G154 suitable for high irradiance exposures of plastics [36]. The combination of UV and condensation at elevated temperatures creates optimum conditions for the accelerated ageing of surfaces. According to ASTM G154, QUV was used to subject the composites to cyclic UV/condensation, thus studying the potential degradation from UV radiation and water condensation by reproducing damage caused by sunlight, rain, and dew. Eight fluorescent UV lamps generate UV radiation in the UV-A region (340 nm). Solar-Eye irradiance detectors monitor and control the intensity of UV radiation in a continuous way. A water bath was used to generate vapor to provide adequate water condensation, simulating dew. This scheme was composed of a continuous cyclic environment consisting of 8 h of UV-A exposure at 60 °C, followed by 4 h of condensation at 50 °C to complete the alternating cycles. The water provided for the experiment was distilled water. Weathered (W) samples were tested in total over a full period of 1000 h.

### 2.4. Nanoindentation Testing

Nanoindentation was performed using a Hysitron Triboscan^®^ Instrument (Hysitron, MN, USA) equipped with a Berkovich diamond tip. Load ranged from 1 μN to 30 mN, while load and displacement resolution were 1 nN and 0.04 nm, respectively. A trapezoidal protocol was adopted [37], with displacement control set at 200 nm to comply with mapping requirements according to ISO 14577-1 standard [38] and ASTM E2546-15, and holding time of 3 s was applied to minimize creep contribution within the unloading curve. E_r_ and H were measured by applying the Oliver–Pharr model within the region of 75–95% of applied load for the unload curve [9,31,39].

**Figure 2:**
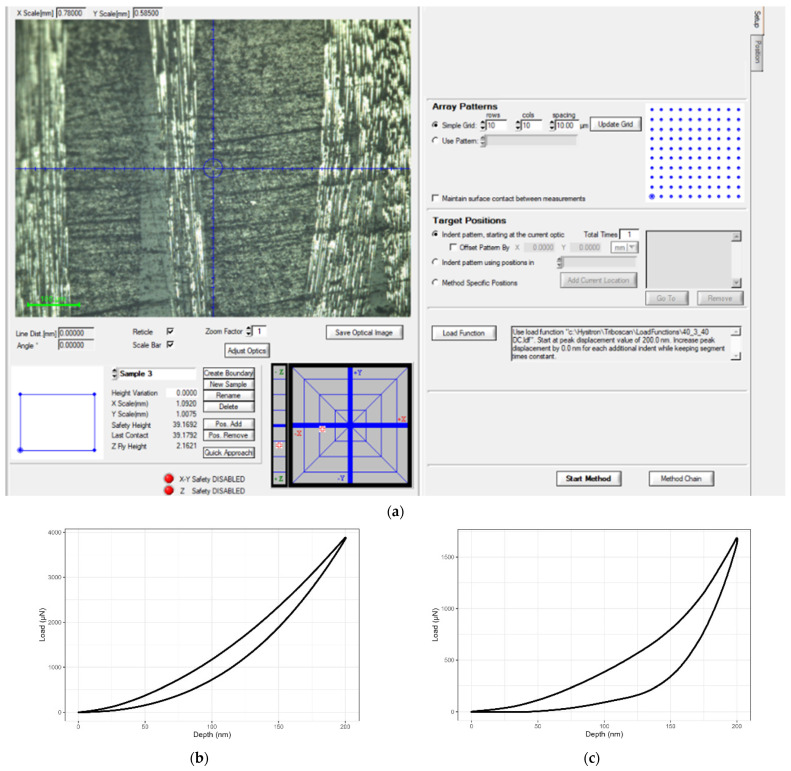

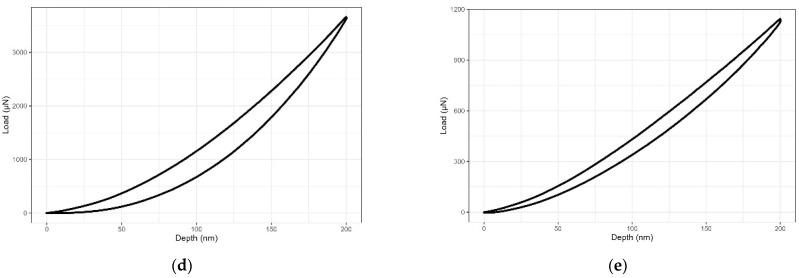
(**a**) Nanoindentation software environment and the mapping grid 10 × 10 used to characterize each specimen, (**b**–**e**) representative load–displacement nanoindentation curves.

In Figure 2, the software environment of nanoindentation is depicted. The indenter probed the material in a square grid of 100 indents (selected area of 90 × 90 μm^2^, 10 μm resolution between indents) in a constant displacement mode of 200 nm. The selection of low penetration depth for mapping was in accordance with literature and a rule of thumb for high-resolution identification and mapping of CFRP phases [6,23,40]. Specimens were polished with a Struers LaboPol-2 grinding machine (Struers Inc., Cleveland, OH, USA). Surficial roughness was reduced using a sequence of SiC grinding papers with 400, 1000, 1200, 2000, and 4000 grit, and Al_2_O_3_ pastes of 3 and 1 μm.

### 2.5. Artificial Intelligence

R language was used to perform analysis with deep learning to obtain insight from nanoindentation data. Data were standardized prior to training and testing in order to avoid bias that may have been induced by the different magnitude of the used data [31]. The pre-processed data were analyzed with deep learning, using an unsupervised method called neural network clustering. Neural networks were chosen since they demonstrate excellent learning ability of complex patterns [31]. An autoencoder was used, while the reproducibility of results was granted by the utility of the used module. A three-layered neural network was developed with 10 neurons in each hidden layer. Tanh was the chosen activation function, while the epoch term, which indicates the number of passes through the entire training dataset, was set equal to 100. All computations were performed using 64-bit Windows 10 Home (Intel^®^ Core™ i5-8250U CPU @ 1.60 GHz, 1801 MHz 4 Cores, 8 Logical Processors and 8.00 GB RAM).

## 3. Results and Discussion

### 3.1. Effect of Modification and Weathering—Mapping of Nanomechanical Properties

Probability distribution analysis (PDA) was performed by applying the normal Gaussian distribution for fitting the histograms of reduced elastic modulus for each specimen tested. The number of phases is identified by histograms, the optimum solution is obtained by incorporating an expectation-maximization algorithm [41], and the volume fraction is quantified. In this way, the outcome of nanomechanical mapping is assessed for possible bias introduced by the local packing density. The normal distribution Gaussian fitness function is presented below:

(1) $$\mathrm{PDF}=\frac{1}{\sqrt{2\pi }\cdot \sigma }\exp\left(-\frac{{\left({Ε}_{r}-\mu \right)}^{2}}{2\sigma^{2}}\right)$$

where E_r_ is the independent variable in Equation (1) and accounts for reduced elastic modulus in GPa, and μ and σ account for the mean value and the standard deviation of each individual phase, respectively.

**Figure 3:**
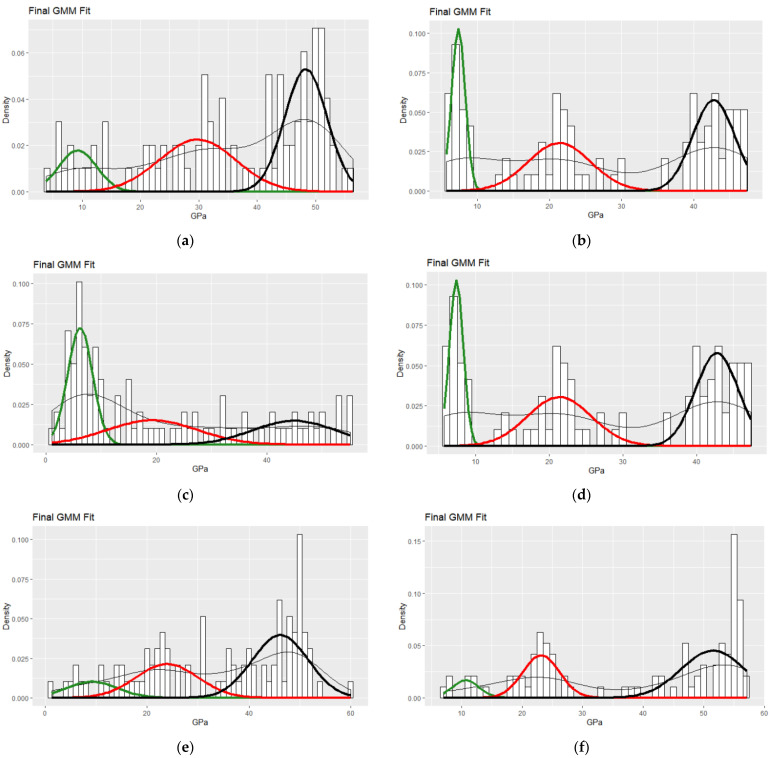
Phase quantification with PDF fitting of nanoindentation-reduced elastic modulus for (**a**) pristine, (**b**) W-pristine, (**c**) PMAA, (**d**) W-PMAA, (**e**) APP, (**f**) W-APP specimen; green: epoxy matrix, red: interface, black: carbon fibers.

In Figure 3, the fitting procedure is based on E_r_ derived from the Oliver–Pharr model [39]. Subsequently, the identification of three CFRP phases was performed by fitting Gaussian curves on the histograms of E_r_ and the results are summarized in Figure 3. The matrix phase is attributed to the first constituent, the epoxy matrix. The second constituent phase is the interface region, and a third is CFs with the higher modulus values.

The unweathered specimens in Figure 3a,c,e demonstrated deviation in E_r_, especially in the case of PMAA electropolymerization reinforcement, where deterioration of nanomechanical properties was evidenced. However, as revealed by PDA (Figure 2, Table S3) this was merely attributed to the packing density (density of fibers in the testing region), which was proved to be lower in the case of the PMAA electropolymerized specimen: 27.8% CF phase versus 48.2 and 54.5% for pristine and APP-treated CFRPs, respectively. Furthermore, the mean E_r_ values of about 44, 46, and 48 GPa for CF phase of these specimens were in accordance with the expected values in literature [7,9,17].

Referring to the reference sample, ageing severely affected the pristine specimen as demonstrated by the weakened interface region. More specifically, the interface E_r_ was reduced by 8.4 GPa after 1000 h of ageing and equal to 21.4 GPa. Furthermore, CF E_r_ mean value was reduced by 5.5 GPa, even though the matrix phase did not demonstrate any drop in modulus; changes in values did not exceed the standard deviation. Thus, the decrement in nanomechanical properties was related to interface/interphase damages due to the propagation of multiple ageing reactions that weakened the composite, namely, chain rapture of the fiber–matrix bond, oxidation, and UV- and thermo- lysis, due to ageing-induced heat fluctuation by weathering as reported in literature [24,42]. Otherwise, the mean value of CF phase would have been higher, if the crosslinked interphase network was not affected.

In the case of functionalized CFRPs in Figure 3c–f, specimens of PMAA electropolymerization and APP treatment exhibited improvement of nanomechanics after weathering treatment. The gain in both cases was that the interface and CF nanomechanical response was uniform, which is demonstrated in the maps by the observation of continuous and interconnected regions; this is indicative of the ability to transfer the load uniformly. In association with the pristine-specimen properties after weathering, the interface E_r_ mean value was higher in both cases of surface modification (Table S3). APP modification revealed enhanced resistance to ageing since interface E_r_ demonstrated no degradation at all after 1000 h of weathering. The effect of enhanced durability compared to PMAA modification and pristine specimens in the case of APP is hypothesized to be attributed to a post-curing-like effect to the enhanced interfacial bonding network of the epoxy–CF system, due to exposure at a temperature range below glass transition during weathering. This effect is combined with the reduction in centers for initiation of depolymerization reactions [43], and reduced penetration of humidity, which are possible explanations for improved elastic properties after weathering.

### 3.2. Effect of Modification and Weathering on Defect Density of CFRPs via Weibull Analysis

Exposure to mild temperatures, humidity, and UV irradiation (weathering protocol) can result in formation of unforeseen defects in the structure, which cannot be detected with conventional characterization tools (Supplementary Materials: microtomography). Defect density may be a key indicator towards higher durability. With chemical modification on the CF surface, it is possible to control the defect density, due to post-curing and crosslinking reactions’ propagation, especially in the interface region. Regarding nanoindentation and Weibull analysis [44], m exponential factor is connected to defect density and is increased by a reduction in the scattering of the imported values. Consequently, it can be a qualitative estimator of the intrinsic structure of CFRPs. m value is measured according to the Weibull distribution function [45]:

(2) $$p=1-e^{\left(-\frac{E_{r}}{E_{\mathrm{ch}}}\right){\;}^{m}}$$

or equivalently,

(3) $$\ln\left(\ln\left(\frac{1}{1-p}\right)\right)=m\cdot (\ln\left(E_{r}\right)-\ln\left(E_{\mathrm{ch}}\right))$$

where p corresponds to survival probability, m is the Weibull modulus, and E_ch_ is the characteristic value of the given parameter E_r_ variation. Both m and E_ch_ are measured after least squares linear regression fitting, and are important parameters that can be used as implications for design. m is connected to uniformity of the specimen regarding the specific property, whereas E_ch_ represents the characteristic value, which represents the indentation modulus with 63.2% probability of occurrence.

As indicated in Table 1 regarding the characteristic E_r_, the epoxy matrix of APP-treated CFRP property is expected to be 30.6% higher than the neat CFRP, and this occasion has a 63.2% probability of occurrence. Even after 1000 h of weathering, this value was higher than the weathered neat CFRP after the same duration by 126.2%, while it also exceeded by 14.3% its previous condition. This is indicative of robust adhesion for APP specimens, and thus such a high matrix modulus value was derived. This is in accordance with the literature, as elastic properties can be highly affected by the fiber-constraint effect [46], which is a reinforcement mechanism for the composite. The Weibull modulus rose to almost 3.3, which is indicative of low scattering of these values around to the characteristic value, which was equal with 22.42 GPa. PMAA modification proved to have a higher E_r,cr_ as regards to the interface, but after weathering this value was comparable with the pristine specimen; however, it is worth mentioning that m was higher by 55.7% after weathering, which was related to higher reliability at service conditions, but yet was not the highest value. In this case, again the APP-treated specimen after weathering demonstrated the highest characteristic value for the interface, which exceeded by 60.3% the pristine property after 1000 h, and at the same time the Weibull modulus obtained the highest value equal with 9.65. The high values of m could be correlated to less heterogeneous microstructures, including the matrix phase of epoxy, and reinforced interface between epoxy and carbon fibers with variations in density [44]. Again, in the case of the pristine specimen, the larger scattering (lower m for the interface) after ageing can be attributed to corrosive degradation of the matrix, and consequently interfacial/interphasial debonding [1,2,5,24].

The reinforcement induced by CFs is largely dependent on adhesion with the matrix phase, and this fact indicates that m values are highly dependent on the interphase ageing behavior, and may lead to different observations for CF-phase m variation. For instance, the CF phase is expected to remain intact after weathering, since turbostratic graphitic structure is corrosion resistive and the hydrophobic nature does not allow water-induced degradation phenomena to occur [47]; however, interphase is not, and in this case chemical modification is expected to reduce humidity penetration, which is the most anticipated corrosive factor, and offer higher durability in the application environment.

In the case of the pristine specimen, m changed from 15.2 to 18.9 for the CF phase, due to weathering (Table 1), but the characteristic E_r_ value was reduced, following the fact that affinity with the epoxy matrix had been deteriorated. This observation is further supported by the decrement of interface m values upon weathering, from 5.91 to 4.94, so that the observations for the CF phase were more consistent. As a result, the distribution of measurements was reduced by 10.7% regarding the characteristic indentation modulus compared to the pristine condition. In the case of the PMAA-treated specimen, the Weibull modulus m was reduced for the CF phase without any significant deviation after ageing reactions for the characteristic modulus. Such change can be attributed to increased defect density [44], for the microstructures that contain both CFs and interphase.

Again, in this case APP treatment demonstrated the optimum performance at service conditions, as simulated by exposure to weathering factors for the full duration of 1000 h, where regarding the CF phase the highest m value was evidenced after weathering, equal to 17.1, which indicates high reliability. Moreover, its characteristic value increased by 9.1% compared to its prior condition, due to post-annealing reactions that occurred upon exposure to repetitive hydrothermal, UV irradiation cycles, which are also connected to increment of Weibull modulus m [44]. Furthermore, E_ch_ was 24.3% higher compared to the weathered pristine specimen. Thus, APP surface treatment of carbon fibers seems to be beneficial to engineer CFRPs resistive to ageing-induced degradation.

### 3.3. Effect of Modification and Weathering on Impact Strength of CFRPs

The observation of the deformation energy at maximum displacement induced by the indenter is connected to the material’s resistance to elastoplastic deformation [48]. This relation is presented below:

(4) $$\frac{H}{E_{r}}=C\cdot \left(\frac{W_{\mathrm{tot}}-W_{u}}{W_{\mathrm{tot}}}\right)$$

where, W_tot_ corresponds to the total work created by the indenter, W_u_ corresponds to the work transferred by the sample to the indenter during unloading, and C is a constant possibly dependent on the indenter angle.

In Equation (4) the numerator is the irreversible work, so that the impact resistance of the tested specimens can be directly related to the nanoindentation hardness and modulus via a linear relation. As the hardness-to-reduced elastic modulus ratio is inversely proportional to the plasticity of a material, it is possible to assess a composite’s tribological durability regarding the impact resistance [49].

Interfacial modification proved thus far, a reinforcing element in CFRPs to enhance nanomechanical properties or reduce the defect density in CFRPs; therefore, a reduction in H/E_r_ ratio was expected upon ageing for composites with a modified interface and increment for pristine composites, due to the availability of a larger surface area for the propagation of decomposition reactions.

**Figure 4:**
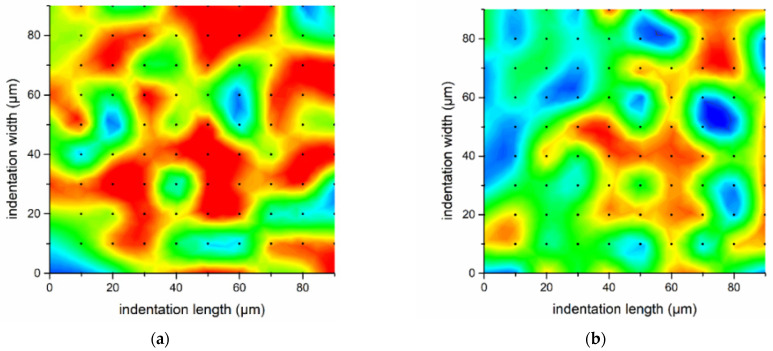

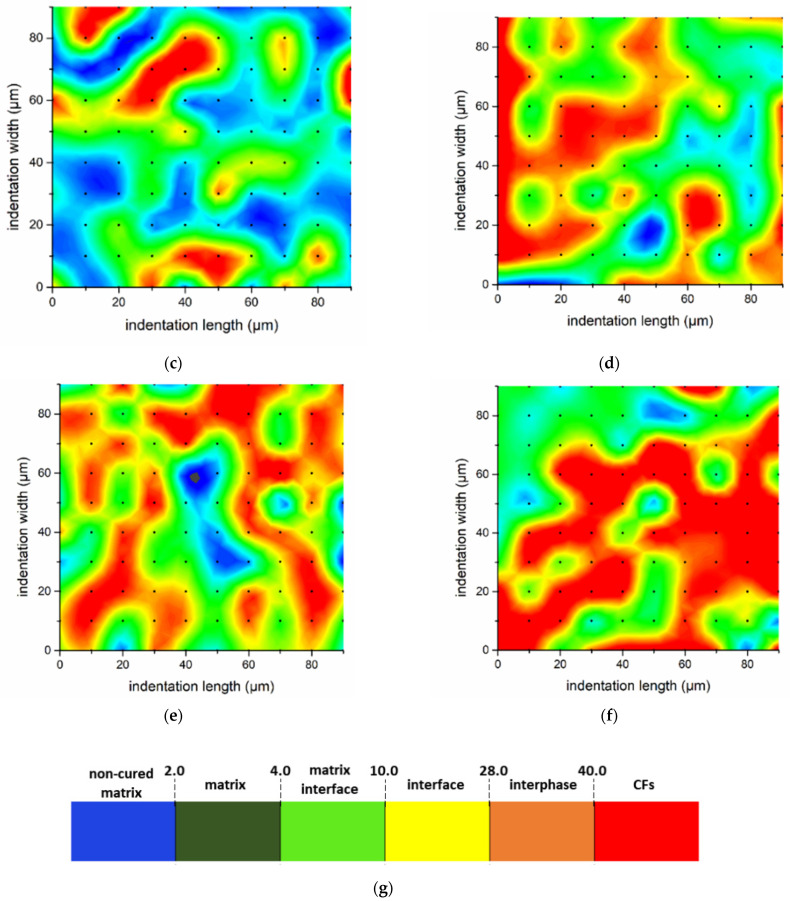
E_r_ contour maps of CFRPs: (**a**) pristine, (**b**) W-pristine, (**c**) PMAA, (**d**) W-PMAA, (**e**) APP, (**f**) W-APP specimen. The memo of the contour maps is presented in (**g**).

In the case of the pristine specimen, two effects of weathering were identified (Figure 4a,b): (a) the epoxy-matrix phase was split into two plasticity zones, as a result of plasticization of the matrix, and (b) CF values were shifted to the right, which was because of lower plasticity obtained in the interface region. The reduced resistance to plastic deformation due to humidity-induced plasticization effects is often responsible for such a reduction [47]. In order to facilitate observation of the results, the reduced elastic modulus contour plots in Figure 4 provide the quantified E_r_ mapping of the tested specimens. The color gradients are in accordance with [9]; the lower E_r_ is indicated in blue color and progressively the red color is reached by the increment of this property.

For PMAA CFRP, the weathering treatment proved to solely affect the epoxy matrix, and not the interface. More specifically, H/E_r_ was increased, which was connected to decrement of matrix plasticity. A similar observation was evidenced regarding the plasticity of the interface/interphase of APP specimens; it was not affected by weathering (Figure 4c,d), but in the case of epoxy matrix, two distinct zones were present with different plasticity values. It is worth mentioning that the second plasticity zone of the epoxy matrix obtained H/E_r_ values in the range of 0.12–0.15. These values were higher in the case of both the pristine and APP specimens compared to PMAA CFRPs, which demonstrated an intermediate median value at 0.10 after weathering and a single homogeneous zone of plasticity. This may be attributed also to the high-volume fraction of CFs in the indented area, and the improved adhesion of the grafted molecular chains of PMAA on the functionalized CF surface.

Ageing decreased durability of epoxy when carbon fibers did not demonstrate high chemical affinity with the epoxy matrix. In APP and PMAA treatments, the ratios in the epoxy matrix were equal to 0.15 and 0.10 (Figure 4e,f), which are regarded as indicative of high durability under service conditions, according to the literature [38]. Thus, it was revealed that PMAA interfacial treatment had a higher potential to extend the life cycle of CFRPs and provide homogeneity and durability, when high-impact resistance was required. Another significant finding was that the clusters that contained E_r_ and H values that corresponded to the interface and CFs demonstrated an increment of H/E_r_ ratio upon ageing propagation in the pristine specimen. On the contrary, APP and PMAA modifications shifted at lower ratio values, and thus higher plasticity. These values were comparable to the condition before ageing or were even better. Consequently, it can be concluded that improvement of nanomechanical properties in aged APP and PMAA-treated CFRPs is a result of increment in plasticity, which functions as a stress-relief mechanism when an impact loading is applied [38].

### 3.4. Modelling of Composites Fingerprint for Condition Monitoring with Artificial Intelligence—Anomaly Detection

Due to the nature of weathering, that incorporates a multitude of corrosive and erosive factors of ageing, it can be hard to distinguish and quantify the magnitude of the degradation process. This is especially significant when materials are tested upon exposure at service conditions. More specifically, treatment for 1000 h can ultimately change the properties in the interface, starting from the surface, and affect the impact resistance of the tested material. Since nanoindentation is in principle a suitable test to measure the impact behavior, instant-hardness monitoring during the loading with the Berkovich tip can provide such information. In detail, upon loading, several transitions can be connected to deformation phenomena, such as pop-in, crack formation, and propagation, which do not correspond to the expected performance of the material upon loading. These defects can be considered as anomalies, since they deviate from normal loading behavior.

In this direction, 500 nanoindentation events were used for each pristine, PMAA, and APP specimen before weathering treatment, feeding machine learning models in a binary classification problem. These data provided the information to train the machine to learn what would be considered as normal data [50], and deploy the trained models to evaluate weathered-specimen condition. The evaluation was performed based on the pristine condition of both modified and unmodified specimens, to compare the effect of weathering prior to and after functionalization in the surface of CFs, while also the model of the unmodified specimen indicated whether a modification should be performed to enhance service-life impact performance. This approach covers both scenarios in case a CFRP should be manufactured using functionalized CFs towards a specific application.

In this case, the contact hardness during loading was used to provide the required data for predictive modeling through machine learning. It is termed as instant hardness [48], as it is a measure of the instantaneous load and the relevant contact area at the respective moment of observation.

(5) $$H_{c}=\frac{F}{A_{c}}$$

(6) $$A_{c}=24.5h_{m}^{2}+{\;a}_{1}h_{m}^{}+{\;a}_{1/2}h_{m}^{1/2}+\ldots +a_{1/16}h_{m}^{1/16}$$

**Figure 5:**
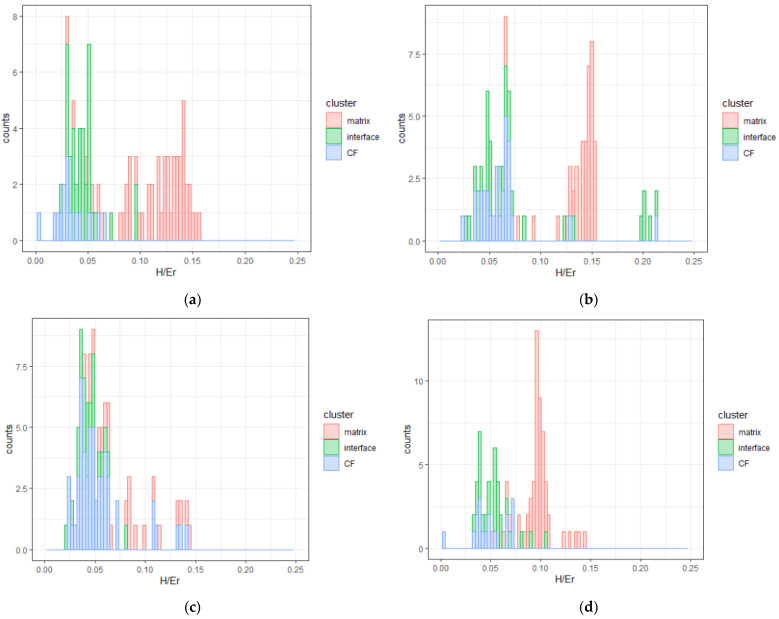

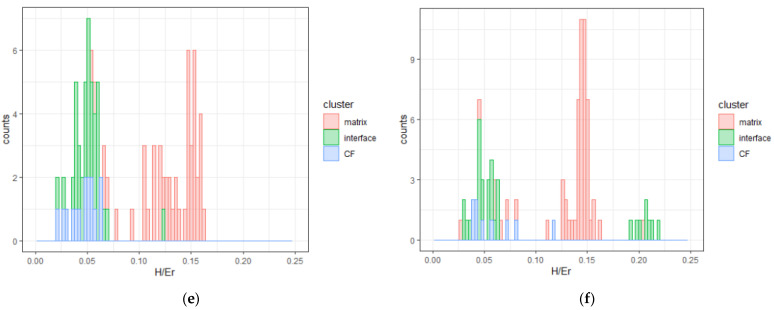
Histogram plots of H to E_r_ ratio for the tested CFRPs. Coloring corresponds to the indented microstructure: (**a**) pristine, (**b**) W-pristine, (**c**) PMAA, (**d**) W-PMAA. (**e**) APP, and (**f**) W-APP.

In this study, H_c_ corresponds to the instant hardness, F is the loading force at the time of observation, and A_c_ corresponds to the instant-contact area, where h_m_ is the indentation depth at the time of observation. For each indentation measurement, load, depth, and time data were involved for the calculation of instant hardness, and nanoindentation strain (h_m_ to pre-set indentation depth ratio). Afterwards, data of instant hardness, nanoindentation strain, and time were retained for each second of nanoindentation-loading trapezoid protocol (1 to 40 s), in a total of 40 observations for each indentation event. Time was retained in the process to train the machine learning models, due to the essential role in the power used for performing the work created by the indenter [49], and thus could feed the machine learning models with patterns relevant to the energy of deformation or the energy retained, due to the CFRP impact-resistance efficiency at service conditions. A total of 20,000 pairs of data were used for the training of the three models (pristine, PMAA, APP) in order to determine what were considered normal data, while 4000 pairs of data were used for the testing dataset (83.3% training and 16.7% testing). In Figure 5, the results of anomaly detection on weathered CFRPs are summarized, regarding the data obtained by nanoindentation.

**Figure 6:**
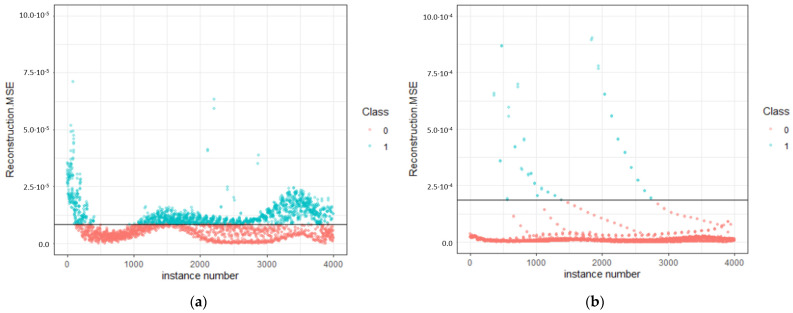

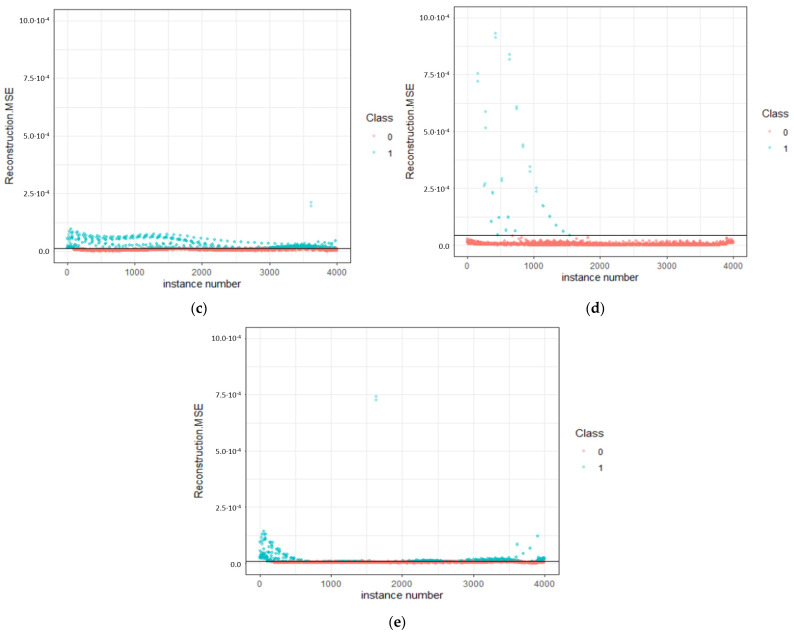
Predictions of (**a**) W-Pristine, (**b**) W-PMAA, (**c**) W-APP on the model trained with pristine data. In (**d**,**e**), the PMAA and APP specimens have been tested by modeling on the respective pristine-specimen data, respectively. The black line corresponds to reconstruction mean square error baseline for classification. Class 0: normality, 1: anomaly.

The model was built to detect patterns in the dataset using the autoencoder function of R H_2_O package in order to identify anomaly data. These values corresponded to degradation due to weathering, whereas the encoder demonstrated the functionality to detect anomaly even in the present case, in data without labels. The architecture of the neural network was complex, including 3 hidden layers consisting of 10 nodes each. The unsupervised model was run for 600 epochs to train the model and predict the normal data as “0” and identify degradation data as “1”. The activation function was the function tanh, and in order to compare the data, mean square error (MSE) was used, and the data were plotted accordingly (Figure 6), which was used to differentiate the cases of environmental exposure-induced degradation. It is evidenced that cases that had higher MSE values (in blue) could be easily distinguished among the data that presented similar MSE values.

This data-driven approach provided insight on the composite fingerprint. This result was an estimator, that correlated in an unsupervised manner the predictors of the features for those three specimens. In Figure 6, the reconstruction mean square error was plotted for each instance, as the measure to classify anomalies. The full code is available at GitHub [51]. This was the output of the model application on the test dataset, and was the measure that identified unexpected behavior as a result of weathering. In Table 2, the abnormal observations are summarized for each model.

In fact, fitting performance was complementary to the % anomalies. The introduced procedure was classified to unsupervised artificial-neural-network clustering. A pattern of data was developed, considered as normal. This means that the fitting of the “normal” data (one cluster) was very accurate. The projection to the new data contained a threshold (value) line/intercluster distance responsible for sorting the normal data out of the failure data (considered as “anomaly”), like a binary classification problem. There was no fitting of a statistical model, but a network built with neurons that learned the pattern. The built network was a feed-forward multilayer artificial neural network, which functioned as a black box, where complex relations were established, and no vision of the functions adopted during training could be obtained, because it is a subject beyond statistics in the field of machine intelligence. More details regarding the learning algorithm can be found at GitHub [51].

In the case of plasma modification, nanoindentation analysis was supported with quantitative evidence by mapping, which exceeded those of the pristine specimen after ageing, and consequently degradation did not occur. In fact, neural networks’ unsupervised clustering identified an effect on the impact resistance. Ageing reactions affected the impact resistance of both pristine and APP specimens after ageing. Testing on the same reference model as a basis for comparison, the pristine specimen demonstrated 44.6% degradation after weathering, while plasma modification reduced the degradation to 25.5%. As a result, it can be concluded that, by modifying the surface of CFs, it is possible to reduce the ageing reactions’ active degradation by almost 20%, for the studied timeline of 1000 h (42 days).

In order to rationalize the effect of weathering and modification of CFs, it is important to summarize the correlated decomposition mechanisms studied in the research field of fiber-reinforced epoxy composites. These fall within the general categories of hydrolysis, thermolysis, and radiolysis, that induce debonding in the matrix, the interfacial region, and the fiber–matrix bond [8,15,20,26]. Degradation phenomena (ASTM G154 [36]) include several corrosive aspects, such as plasticization, due to humidity diffusion in the matrix and UV irradiation-induced debonding, and microcrack generation, due to the composite exposure to thermal cycles including constituent phases with different thermal expansion coefficients (fiber and matrix). The plethora of these activities may not be prevented by supporting the crosslinking of CFs with epoxy resin by the addition of oxygen functional groups alone. This is the reason why, even when comparing the weathered APP specimen based on the model trained with APP CFRPs data, 24.0% of data were classified as anomalies connected to reduction in the resistance to plastic deformation.

PMAA sizing of carbon fibers demonstrated an optimized behavior regarding plastic deformation after weathering. The grafted PMAA has the uniqueness that molecular weight of grafted macromolecules cannot be precisely controlled, but demonstrates a statistical variation amongst fiber length. Therefore, it is possible that several different zones are formed initially and interfacial reinforcement is induced by the roughness of the PMAA different-length chains’ brush-like structure at the nanoscale level as previously studied [52]. This stereochemistry may be beneficial in the stress release of the stress created due to the different expansion coefficients, by offering a wider interface, which allows for prevention of crack formation and propagation, and consequently facilitates the minimization of active surfaces for ageing-induced corrosion. Moreover, chemical affinity of surficial chemistry of the fiber and matrix in the interface region can be transformed to chemical bonding upon exposure to several thermal cycles during exposure at service conditions. These intrinsic changes are expected to enhance homogeneity of reinforcement zones by post-curing reactions [3,24]. This was confirmed with machine learning, which demonstrated a minor degradation of 2.9% based on the model trained with pristine data, while it became equal to 1.8%, when using PMAA CFRPs for training, which could be considered as statistically insignificant compared to the weathered pristine and APP specimens.

## 4. Conclusions

Nanoindentation was used as a primal characterization tool to evaluate chemical modification of carbon-fiber surfaces. Fast assessment and high-resolution mapping enabled microstructure quantification by performing probability distribution analysis. Nanomechanical properties of CFRPs demonstrated improvement in ageing behavior of CF phase upon 1000 h exposure to UV irradiation and heat applied by simulating real application conditions with weathering, due to plasma and electropolymerization engineering of CFs.

Characteristically, weathering severely affected the pristine specimen with reduction in the interface elastic properties by 8.4 GPa after 1000 h of ageing. Additionally, CF indentation modulus was decreased by 5.5 GPa, as it was influenced by interface corrosion due to the effect of UV-, thermo-, and hydro-lysis reaction propagation, owed to the conditions of the weathering protocol. In the case of PMAA, interface properties were increased by 13% after weathering. APP functionalization demonstrated the highest indentation modulus after weathering, equal to 54.92 GPa with a 63.2% probability of occurrence according to Weibull analysis. Even after 1000 h of weathering, this value was higher than the weathered neat CFRP by 126.2% in the case of the epoxy matrix for the same duration of exposure, while it also exceeded by 14.3% its previous condition. Such improvement is hypothesized to be associated with the reduction in active sites for ageing-induced degradation, as a result of the improved wetting of the fiber and matrix; thus, matrix properties are highly influenced by the carbon-fiber elastic properties.

Additionally, H/E_r_ ratio demonstrated a plastic mechanism for stress relief upon (nano-)impact loading. The low H/E_r_ ratio was indicative of increased durability for application at real service conditions. This is a beneficial effect, which is obtained by the engineering of the interface by modifying the surface chemistry of CFs.

Some additional insights were obtained by complementary characterization (Supplementary Materials). Micro-CT X-ray imaging proved that exposure of CFRPs to open-field conditions did not lead to the increment of porosity. Still, after weathering, total porosity accounted for a magnitude of 10^−2^ percentage (0.01%), which is nearly zero (statistically insignificant) total porosity. A degradative layer was evidenced on the surface, with penetration depth in the magnitude of tens of micrometers.

Finally, artificial intelligence was incorporated for condition monitoring of CFRPs upon modification and weathering. Unsupervised machine learning was implemented via deep learning, by using neural networks with three layers for processing the data of impact strength. Training data included 20,000 observations from nanoindentation analysis of as-fabricated specimens, in order to identify normality and ageing-induced anomalies after modification and weathering. In this case, anomaly detection demonstrated that PMAA modification contributed to retaining resistance to plastic deformation after weathering, compared to the pristine and APP specimens; in the latter cases, anomalies accounted for 44.6% and 24.0%, respectively. Thus, both modifications are expected to improve CFRP impact resistance under service conditions and improve the reliability of CFRPs.

## Figures and Tables

**Table 1:** Weibull modulus m for the tested CFRPs for E_r_.

	Matrix		Interface		CFs	
	m	E_r,ch_ (GPa)	m	E_r,ch_ (GPa)	m	E_r,ch_ (GPa)
Pristine	2.31	14.71	5.91	36.75	15.20	49.47
PMAA	1.88	11.01	5.56	42.14	14.30	51.27
APP	1.80	19.21	7.67	37.02	13.50	50.33
Weathered pristine	3.30	9.91	4.94	26.56	18.90	44.18
Weathered PMAA	2.00	13.26	7.69	25.15	6.89	51.07
Weathered APP	3.28	22.42	9.65	42.58	17.10	54.92

**Table 2:** Quantified results by deep learning on the weathered specimens.

Model & Weathered Specimen	Pristine-on-Pristine Model	PMAA-on-Pristine Model	APP-on-Pristine Model	PMAA-on-PMAA Model	APP-on-APP Model
**Anomalies**	1783	117	1020	70	958
**Percentage**	44.6%	2.9%	25.5%	1.8%	24.0%

## Supplementary Materials

The following are available online at www.mdpi.com/article/10.3390/nano11102631/s1, Table S1. Measurement parameters of Sky Scan 1272 CT; Table S2. Results of 3D analysis performed by CTan software; Figure S1. Intensity of Pore Size Distribution; Figure S2. (a) Ageing Depth through reconstructed images, (b) Images of CFRPs pre and post exposure; Table S3. Descriptive statistics of CFRPs phases by implementing PDF on reduced Elastic Modulus data.
