# Supplementary material for: Carbon Fiber Reinforced Composites: Study of Modification Effect on Weathering-Induced Ageing via Nanoindentation and Deep Learning
Source: Nanomaterials (Basel). 2021 Oct 6;11(10):2631. doi: 10.3390/nano11102631 (PMC8539085; doi:10.3390/nano11102631)
Supplement: Supplementary file 1 [file nanomaterials-11-02631-s001.zip › nanomaterials-1316431-supplementary.pdf]

nanomaterials

MDPI

Supplementary Materials

Carbon Fiber Reinforced Composites: Study of Modification Effect on Weathering-Induced Ageing via Nanoindentation and Deep Learning

Georgios Konstantopoulos [1], Dionisis Semitekolos [1], Elias P. Koumoulos [2,*] and Costas A. Charitidis [1]

[1] RNANO Lab—Research Unit of Advanced, Composite, Nano Materials & Nanotechnology, School of Chemical Engineering, National Technical University of Athens, GR-15773 Zographos Athens, Greece; gkonstanto@chemeng.ntua.gr (G.K.); diosemi@chemeng.ntua.gr (D.S.); charitidis@chemeng.ntua.gr (C.C.)
[2] Innovation in Research & Engineering Solutions (IRES), Boulevard Edmond Machtens 79/22, 1080 Brussels, Belgium
* Correspondence: epk@innovation-res.eu

Microcomputed X-Ray Tomography analysis (micro-CT)

Bruker's micro-CT, SkyScan 1272 (Bruker, Kontich, Belgium), was used to receive quantitative information of the internal structure of the samples, by 3D imaging. The system consists of a microfocus sealed X-ray source, which operates at 20–100kV and 10W (<5 μm spot size @ 4W), an X-ray detector with a maximum resolution of 11 MP (4032 × 2688 pixels) and a 14-bit cooled CCD fibre optically coupled to scintillator. The parameters are summarized in Table S1. The composite samples were cut with a water wheel at the required dimensions: 1 cm x 0.7 cm x 0.3 cm. 2D images of cross-sectional slices were reconstructed via N Recon Reconstruction software (Microphotonics, Allentown, Pennsylvania)). CTan software (Blue Scientific, Cambridge, UK) was used to perform the 3D analysis and CTvox to collect 3D images.

Table S1. Measurement parameters of Sky Scan 1272 CT.

Voltage (kV)	Current (μA)	Filter (mm)	Pixel Size (μm)	Resolution
40	166	Al 0.25	9	1344 × 896

Porosity of CFRPs

Micro-computed tomography came up as an essential tool to characterize the preparation method of the composite structure. More specifically, porosity of the indented structures can significantly affect the mechanical properties. In the present case, analysis of porosity was performed in the whole volume of CFRPs samples and is informative even for the porosity alternations owed to the weathering process. CTan software was used to perform the 3D analysis and CTvox (Blue Scientific, Cambridge, UK) to collect 3D images of the scanned samples. Table S2 presents the porosity of weathered and non-weathered composite materials.

Table S2. Results of 3D analysis performed by CTan software.

	Pristine	PMAA	APP	W-Pristine	W-PMAA	W-APP
**Open Porosity (%)**	0.0019	0.0021	0.0010	0.0203	0.0156	0.0115
**Total Porosity (%)**	0.0253	0.0206	0.0132	0.0459	0.0351	0.0244

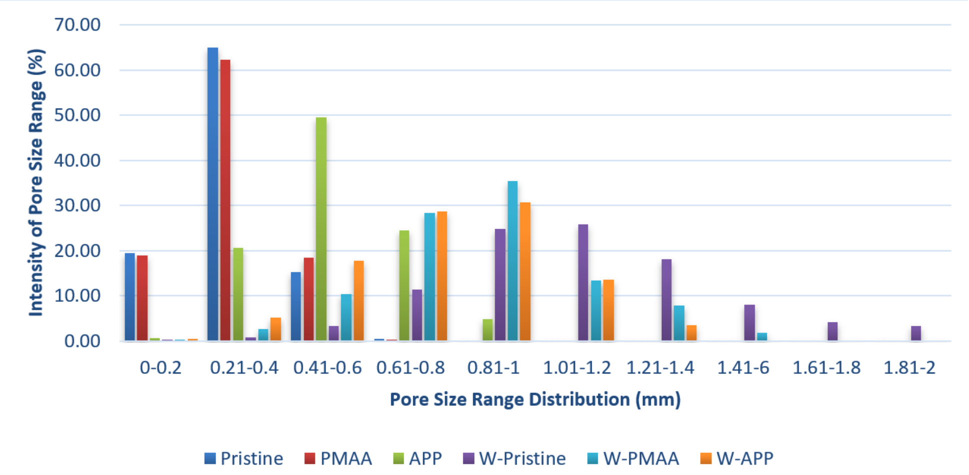

Figure S1. Intensity of Pore Size Distribution.

It can be observed that samples exhibit a low, near zero in value, total porosity, attributed to the vacuum assisted resin transfer molding manufacturing process. The changes in porosity attributed to the accelerated weathering process seem to affect the weathered samples by a factor of two, resulting in a maximum value of 0.046% for the pristine sample. This value was lower for the modified specimens. More specifically, open porosity is increased after weathering, due to the degradation of the surface epoxy layer. Figure S1 illustrates the percentage for each pore size range. It is worth to mention that the pore size distribution demonstrated a right shift after the weathering cycling protocol. For the same weathered samples, the size reaches a maximum of 2 mm for the composite with pristine fabric, indicating that weathering conditions escalated within the defective sites. However, it should be mentioned that the generation of pores with higher diameter corresponds to 0.046 %, 0.035 %, and 0.024 % for pristine, PMAA-modified, and APP-modified CFRPs, respectively. It can be observed that, for samples prior to exposure in accelerated weathering conditions, the size of their pores ranged between 0.2 mm to 0.6 mm.

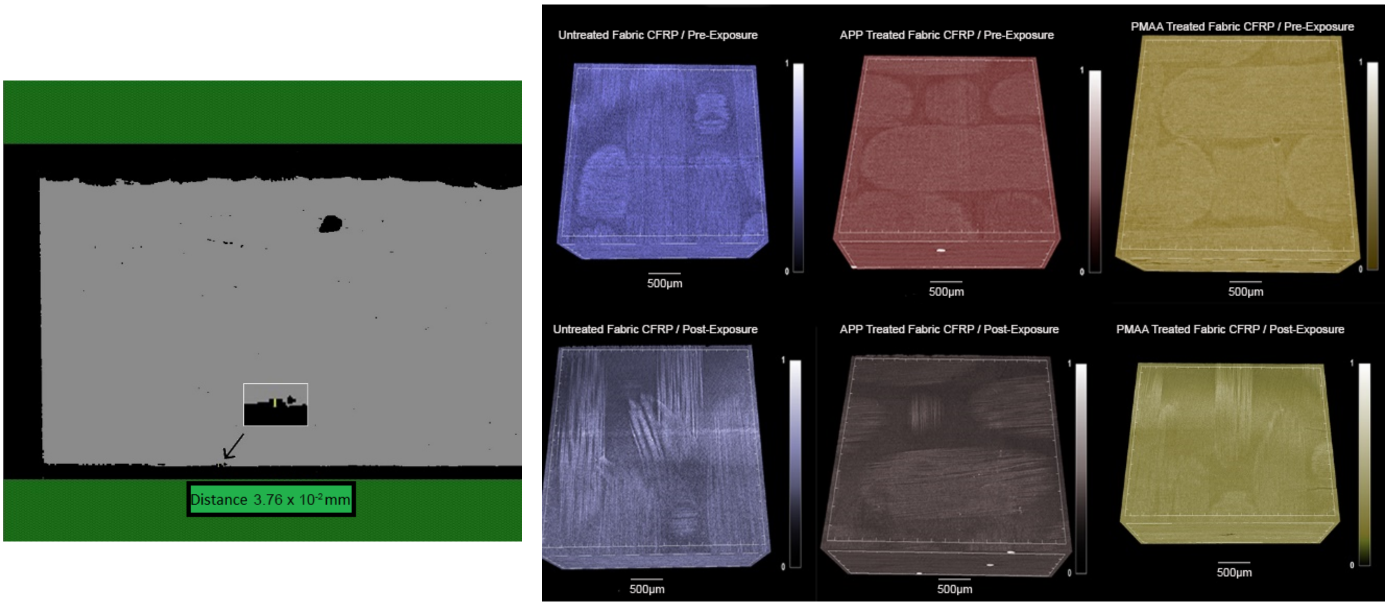

(a) (b)

Figure S2. (**a**) Ageing Depth through reconstructed images, (**b**) Images of CFRPs pre and post exposure.

An estimation of the ageing depth of CFRPs pre- and post- exposure can be evaluated through the reconstructed images as depicted in Figure S2. By taking advantage of CTan software the magnitude of the surface degradation can be assessed. Depth ranges from 30 μm to 70 μm have been observed for the aged composites. Additionally, images confirm the mere surficial epoxy layer degradation. Images of the CFRPs after the exposure in environmental conditions appear to be glossier than of those not exposed.

Mapping of Nanomechanical Properties

Table S3. Descriptive statistics of CFRPs phases by implementing PDF on reduced Elastic Modulus data.

	matrix			interface			CFs		
	PDF mu	PDF si	Volume Fraction	PDF mu	PDF si	Volume Fraction	PDF mu	PDF si	Volume Fraction
Pristine	9.25	2.26	14.6%	29.80	6.59	37.2%	48.34	3.64	48.2%
PMAA	6.19	2.31	41.1%	19.27	8.16	31.0%	44.93	7.46	27.8%
APP	9.21	5.00	12.8%	23.92	6.08	32.6%	46.18	5.50	54.5%
W-Pristine	7.38	0.95	24.6%	21.39	4.35	33.2%	42.79	2.92	42.2%
W-PMAA	10.67	4.74	14.5%	21.72	3.17	24.2%	46.23	9.31	61.3%
W-APP	10.67	2.21	9.3%	23.14	3.04	31.1%	51.52	5.25	59.6%

In Table S3, the detailed calculations are summarized regarding Probability Distribution Analysis. Upon PMAA treatment the matrix and interface properties significantly decreased compared to the pristine specimen by 33 and 35%, respectively. In case of APP treatment, interface properties decreased by 20%, while matrix and CFs properties varied within standard deviation of the pristine specimen.

Even though modification of CFs did not provide measurable improvement initially, it is worth investigating the alternations that were introduced and the resistance to the ageing degradative mechanism induced by exposure to weathering conditions. In case of the pristine specimen, weathering reduced the E_r mean value of the matrix phase by 20%. For both functionalization approaches, epoxy matrix demonstrated a slight increment in E_r up to 10.7 GPa, which in case of PMAA falls within the standard deviation compared to the aged pristine specimens, and is comparable to the pristine specimen matrix phase.

This increment may not be originated by epoxy post-curing reactions, due to the exposure to weathering factors, but is more probably connected to the enhanced mechanical interlocking with carbon fibers; considering the fact that fiber constraint effect is affecting the matrix properties in a higher extend compared to the specimens prior to exposure. In the case of PMAA, it is worth to mention that interface properties were increased after weathering by 13%, while in APP no changes are observed, and the obtained values in both cases are comparable to weathered pristine specimen.

The elevation of nanomechanical properties in case of APP composite, outperforming all specimens, can be connected to improved mechanical interlocking due to plasma functionalization. The low penetration depth of Plasma irradiation on carbon fibers surface could prevent the oxidative degradation effects and introduce new chemical functionality and microporosity [1].and improve the composite nanomechanical properties [2]. Apart from mechanical robustness obtained by plasma modification, enhancement of nanomechanical properties is an implication of improved wetting, as proved by hydrothermal durability during ageing [3] is connected to reduction of the available interfaces for ageing-induced degradation.

References

1. Jones, F.R. A Review of Interphase Formation and Design in Fibre-Reinforced Composites. *J. Adhes. Sci. Technol.* **2010**, *24*, 171–202, https://doi.org/10.1163/016942409x12579497420609.
2. Sun, J.; Zhao, F.; Yao, Y.; Jin, Z.; Liu, X.; Huang, Y. High efficient and continuous surface modification of carbon fibers with improved tensile strength and interfacial adhesion. *Appl. Surf. Sci.* **2017**, *412*, 424–435, https://doi.org/10.1016/j.apsusc.2017.03.279.
3. Wang, Z.; Xian, G.; Zhao, X.-L. Effects of hydrothermal aging on carbon fibre/epoxy composites with different interfacial bonding strength. *Constr. Build. Mater.* **2018**, *161*, 634–648, https://doi.org/10.1016/j.conbuildmat.2017.11.171.
